# Mother infant zero separation for neonatal jaundice: we are getting closer

**DOI:** 10.1186/s13052-025-02104-6

**Published:** 2025-08-15

**Authors:** Riccardo Davanzo, Paola Cavicchioli, Massimo Agosti, Carlo Dani

**Affiliations:** 1 Nutrition Research Centre, University of Insubria, Varese, Italy; 2 Pediatrics & Neonatology Dpt, Ospedale dell’Angelo, Mestre, Italy; 3 Pediatric Department, Hospital ’F. Del Ponte’, University of Insubria, Varese, Italy; 4 Department of Neurosciences, Psychology, Drug Research and Child Health, Careggi University Hospital, Florence, Italy; 5 Division of Neonatology, Careggi University Hospital, Florence, Italy; 6 RIFAM (Italian Network of Trainers in Breastfeeding), Via Navali 28, Trieste, 34143 Italy

**Keywords:** Breastfeeding, Jaundice, Cyclic phototherapy

## Abstract

**Background:**

Although phototherapy represents the standard of care for preventing bilirubin neurotoxicity, it can have both short- and long-term adverse effects. Moreover, phototherapy can interfere with mother-infant relationship and breastfeeding.

**Main body:**

As phototherapy quickly converts the bilirubin in the skin compartment and in the cutaneous circulation into harmless photo-isomers, during the following 2–3 h the effect of phototherapy is limited, leading to the plausibility for an effective intermittent phototherapy, which in fact has been recently documented both in preterm and term neonates.

**Conclusions:**

Cyclic phototherapy can help reduce mother-infant separation to a minimum, thus promoting the development of the mother-infant relationship and, ultimately, exclusive breastfeeding.

**Supplementary Information:**

The online version contains supplementary material available at 10.1186/s13052-025-02104-6.

## Background

Phototherapy (PT) represents the standard of care for preventing bilirubin neurotoxicity, accounting for about 4–5% of newborn infants. Its clinical effects are affected by several parameters: the type of equipment used (i.e.: light emitting diodes versus fluorescent tubes and/or halogen lamps), the spectrum of the light emitted, the level of irradiance, the percentage of skin surface exposed, the distance between the light source and the patient’s skin, and the duration and mode (continuous or intermittent/cyclic) of treatment. Although these parameters vary greatly across studies, making a comprehensive assessment of PT efficacy and safety difficult, there is sufficient evidence that it can have both short- and long-term adverse effects: hypothermia in the absence of adequate thermal control, reduced splanchnic oxygenation and transient feeding tolerance, photodynamic damage with synthesis of free radicals by photo-oxidation, DNA damage, geno-cytotoxicity, cytokine activation and impaired immunity, increased risk of seizures and solid tumors [1].

### Main text

Hospital admission for jaundice requiring PT also causes mother-infant separation which can lead to parental stress and anxiety, hamper the establishment of exclusive breastfeeding or even cause early interruption after hospital discharge [2]. Moreover, according to Kemper et al. mothers of jaundiced newborns submitted to phototherapy were more concerned and possibly insecure about their infant’s overall health at 1-month postnatal age, although this study refers to the experience and neonatological practice of almost 40 years ago when parental counselling and individualized care were less widespread [2].

On the other hand, reducing hospitalizations for phototherapy can help optimize care costs and free up resources for other tasks, including in the middle- and low- income settings. Consequently, trying to get closer to zero separation between mother and baby due to neonatal jaundice is a rational and desirable goal.

The total serum bilirubin (TSB) thresholds for starting PT are detailed in nomograms that account for additional risk factors for neurological damage, such as gestational and postnatal age, hemolytic pathologies, sepsis, and significant clinical instability in the previous 24 h [3]. The 2022 guidelines of the American Academy of Pediatrics on the management of hyperbilirubinemia increased the threshold for starting PT of about 2 mg/dL [3]. Consistently, the implementation of these guidelines [3] allowed to significantly decrease the number of TSB measurements and halved hospital admission for PT to about 2% without increasing readmission for jaundice in infants born at *≥* 35 weeks of gestation.

In current practice, PT is usually applied continuously, and its suspension occurs upon achievement of TSB goals. Nevertheless, cycled/intermittent PT with periods of non-exposure has been used since the 70ies. PT is defined as cyclic when the exposure lasts a few minutes (i.e.: 15–30 min) per hour while is defined as interrupted or intermittent when exposure lasts 1–2 h and alternates with its suspension for at least one hour (for example: 1 h on and 2 h off) and used after the initial decline in bilirubin. The effectiveness of interrupted and cyclic phototherapy can be explained by its mechanism of action. Initially, bilirubin in the skin compartment absorbs photons of phototherapy and additional bilirubin migrates from the blood to the skin, but this migration occurs slowly (2–3 h) and during this time the effect of phototherapy is limited. Therefore, it is rational to interrupt phototherapy for the time necessary for bilirubin migration and then resume it. Recent studies have demonstrated in premature infants that cyclic PT (15–30 min per hour) halves light exposure without affecting the decrease in TSB and without evidence of neurotoxicity [4], while splanchnic and cerebral oxygenation remain stable [5]. Furthermore, PT cycles of 1 h on and 2 h off in neonates with gestational age ≥ 34 weeks have been shown to be effective in reducing its duration by two-thirds and doubling the TSB decrease rate in comparison with continuous PT [5]. On this regard, AAP guidelines underline that “*interrupting phototherapy for breastfeeding does not impact the overall effectiveness of phototherapy*” and specify that “*these interruptions should be minimized if the bilirubin concentration is approaching the need to escalate care”*, that is when TSB is 2 mg/dL below the exchange transfusion threshold [3].

Other interventions should complement the care of neonates for preventing and/or treating hyperbilirubinemia. Whenever possible, PT should be managed in the mother’s room or in a room where the mother can stay with the baby and breastfeed [3], possibly resorting to the galactogenic effect of skin-to-skin contact. In fact, the proximity of the mother to the newborn also allows jaundiced newborns, who do not always spontaneously show feeding cues, to be woken up at least every three hours, as breastfeeding fewer than 8 times per day has been associated with higher incidence of hyperbilirubinemia also after hospital discharge [6].

On the other hand, when the intake of breast milk is inadequate and infants are dehydrated (i.e.: >8–10% weight loss and/or hypernatremia), TSB can be lowered increasing the number of feedings and supplementing the baby with expressed mother own milk, and/or formula milk. Furthermore, it is advisable to respect the PT thresholds without starting it before having exceeded them, just as it is advisable to avoid inertia in interrupting the PT when it is no longer necessary.

Science and prudence therefore suggest limiting mother-infant separation as much as possible, pursuing this objective right away since we already have guidelines [3] that have been shown to reduce the need for hospital admissions for jaundice. Accordingly, we can encourage mother-child contact by using intermittent/cyclic PT [4, 5] when it becomes necessary. Furthermore, under certain conditions and with caution, PT could be performed at home in neonates who have already been discharged and who then develop a TSB above the threshold and require treatment [3]. This is especially of interest in contexts with limited access to modern PT equipment, e.g., middle- and low-income settings.

## Conclusions

In conclusion, while mother-infant separation can never be completely zeroed, a proactive and comprehensive approach to the management of hyperbilirubinemia using current resources, including cyclic and interrupted PT, can help reduce it to a minimum. Any time, even short, gained to promote parental-newborn interaction, in an ambient light that might allow the baby to open its eyes and to be lovingly hugged should be sought. Protecting the closeness of the dyad may represent a golden goal for neonatal care since it is an inexpensive method to prevent possible adverse effects and promote not only the development of the mother-infant relationship, but also the comfort of the newborn, the reduction of maternal anxiety and, ultimately, exclusive breastfeeding [7].

## Supplementary Information

Below is the link to the electronic supplementary material.

Supplementary Material 1

## Data Availability

Not applicable.

## Abbreviations

PT: Phototherapy
TSB: Total serum bilirubin
