# Supplementary material for: Mother infant zero separation for neonatal jaundice: we are getting closer
Source: Ital J Pediatr. 2025 Aug 15;51:254. doi: 10.1186/s13052-025-02104-6 (PMC12357473; doi:10.1186/s13052-025-02104-6)
Supplement: Supplementary file 1 — Supplementary Material 1 [file 13052_2025_2104_MOESM1_ESM.doc]

*To dr Laura Travan*

*and to Reviewers of the paper*

MANUSCRIPT NUMBER: ITLP-D-25-00169 
AUTHORS: RICCARDO DAVANZO ET AL

*Italian Journal of Pediatrics, Chief Editor,*

*we have read carefully the informative and constructive comments of the reviewers. We are grateful for their appreciations of our paper and for the precious suggestions. Consequently, we have changed our paper as follows:
According to Reviewer 1*

- *We have specified the differences between cyclic and interrupted phototherapy.*
- *We have underlined the interference of phototherapy with mother-infant bonding*
- *We stressed the importance also of a reduction even if limited in time of phototherapy, when not yet necessary*
- *We have added some references to sustain new statements of the current version*
- *According to Reviewer 2
  We have specified the usefulness of cyclic/intermittent phototherapy for low-middle income countries.*

*Best wishes and many thanks, once more!*

*Riccardo Davanzo, MD, PhD*
